# People from ethnic minorities seeking help for long COVID: a qualitative study

**DOI:** 10.3399/BJGP.2023.0631

**Published:** 2024-10-15

**Authors:** Nina Smyth, Damien Ridge, Tom Kingstone, Dipesh P Gopal, Nisreen A Alwan, Alexa Wright, Ashish Chaudhry, Sophie Clark, Rebecca Band, Carolyn A Chew-Graham

**Affiliations:** School of Social Sciences, University of Westminster, London.; School of Social Sciences, University of Westminster, London.; School of Medicine, Keele University, Newcastle-under-Lyme, and Research and Innovation Department, Midland Partnership University NHS Foundation Trust, Stafford.; Wolfson Institute of Population Health, Queen Mary University of London, London.; School of Primary Care, Population Sciences and Medical Education, University of Southampton, and University Hospital Southampton NHS Foundation Trust, Southampton.; School of Humanities, University of Westminster, London.; Faculty of Biology, Medicine and Health, University of Manchester, Manchester, and patient advisor.; NIHR Applied Research Collaboration Wessex, and School of Health Sciences, University of Southampton, Southampton.; NIHR Applied Research Collaboration Wessex, and School of Health Sciences, University of Southampton, Southampton.; School of Medicine, Keele University, Newcastle-under-Lyme.

**Keywords:** ethnic minorities, long COVID, primary health care

## Abstract

**Background:**

People from ethnic minority groups are disproportionately affected by COVID-19, less likely to access primary health care, and have reported dissatisfaction with health care. Although the prevalence of long COVID in ethnic minority groups is unclear, such populations are underrepresented in long-COVID specialist clinics and long-COVID lived-experience research, which informed the original long-COVID healthcare guidelines.

**Aim:**

To understand the lived experiences of long COVID in people from ethnic minority groups.

**Design and setting:**

Qualitative study of people living with long COVID in the UK.

**Method:**

Semi-structured interviews with people who self-disclosed long COVID were conducted between June 2022 and June 2023 via telephone or video call. Thematic analysis of the data was conducted. People who were living with long COVID, or caring for someone with long COVID, advised on all stages of the research.

**Results:**

Interviews were conducted with 31 participants representing diverse socioeconomic demographics. Help-seeking barriers included little awareness of long COVID or available support, and not feeling worthy of receiving care. Negative healthcare encounters were reported in primary health care; however, these services were crucial for accessing secondary or specialist care. There were further access difficulties and dissatisfaction with specialist care. Experiences of stigma and discrimination contributed to delays in seeking care and unsatisfactory experiences, resulting in feelings of mistrust in health care.

**Conclusion:**

Empathy, validation of experiences, and fairness in recognition and support of healthcare needs are required to restore trust in health care and improve the experiences of people with long COVID.

## Introduction

Long COVID, the patient-preferred term,^1^ is the experience of persistent symptoms for ≥ 12 weeks following a COVID-19 infection. In early 2023, long COVID was estimated to affect 1.9 million adults in the UK,^2^ and an estimated 200 million people were thought to be affected globally in 2019.^3^ The National Institute for Health and Care Excellence (NICE)^4^ uses the term ‘post-acute COVID-19 syndrome’. Symptoms can be physical, cognitive, or psychological,^5^ with debilitating impacts on people’s daily lives.^5^^–^7 People living with long COVID have described having difficulty accessing health care or feeling that they are not believed by GPs.^8^^–^12 GPs have also described their own challenges in managing people with long COVID.^13^

People from ethnic minorities were disproportionately affected by the COVID-19 pandemic; this was related to socioeconomic deprivation and working in sectors that were exposed to COVID-19.^6^^,^^14^ However, the evidence on the prevalence of long COVID among ethnic minority groups is conflicting.^15^^–^19 People from these backgrounds are less likely to present to primary health care, as indicated by clinical coding of long COVID,^20^ less likely to gain access to long-COVID clinics,^21^^,^^22^ and are underrepresented in long-COVID research.^8^^,^^23^ In general, people from ethnic minorities have reported less satisfaction with primary health care because of factors including: lack of care demonstrated by practitioners, a lack of trust in professionals, and being less involved in decision making.^24^

People living with long COVID experience stigma,^12^^,^^25^ and are devalued or discredited by society because of their condition.^26^ Stigma is not an uncommon experience for people living with health conditions that have a history of sensationalism in the media, such as mental health difficulties or HIV.^27^^,^^28^ People may experience health-related stigma in different ways, such as through social exclusion, rejection, blame, and negative attitudes associated with their health condition.^29^ Stigma can be further impacted by intersections with pre-existing inequalities, such as a person’s gender, ethnicity, or sexuality.^28^^,^^30^ The damaging impact of stigma on healthcare access and experiences, as well as health outcomes, is well documented.^27^^,^^28^^,^^31^^,^^32^ Stigma experiences may be wide ranging for people living with long COVID from ethnic minority backgrounds; for example, there may be stigma attached to having COVID-19 from others in various community settings^31^ and patients may experience discrimination in health care (for example, related to racism).^33^ All of this may contribute to avoidance of the ‘long-COVID’ label, and disengagement with health care and/or inadequate healthcare experiences, thereby potentially widening health inequalities.^28^^,^^32^^,^^34^

This study aimed to explore the lived experiences of long COVID in people from ethnic minorities in the UK, focusing on their experiences of accessing health care.

**Table:** How this fits in

People from ethnic minority groups are less likely to present to primary health care for long COVID; this study explored the lived experiences of long COVID in people from such groups. Participants were often previously unaware of long COVID or available support, and some described not feeling worthy of receiving care. Experiences of stigma and discrimination contributed to a lack of trust in healthcare professionals and services, and were common when participants had experienced previous negative healthcare encounters. Receiving empathy, validation, and fairness in the recognition of symptoms, as well as support, is needed to enhance trust and safety in health care.

## Method

Qualitative methodology was used to understand experiences of long COVID, enabling an exploration of lived experiences,^35^ which is crucial to informing care and management of health conditions.^4^^,^^36^ Further details can be found in the study protocol written by Smyth *et al*.^37^

### Advisory groups

A patient advisory group consisted of one male and six females who were living with, or caring for, someone living with long COVID, and who had a range of ethnic minority backgrounds. This group complemented an expert advisory group of six healthcare practitioners and health service researchers interested in long COVID and ethnic minority health. Patient advisors helped to develop the project aims, while both groups advised on study design, methodology, analysis, and dissemination.

### Participants

Adults, aged >18 years, with self-disclosed long COVID, were recruited via advertisements through social media, support groups, university sites, faith/religious networks, community organisations, and advisory groups across England. Potential participants contacted the research team and were sent a Participant Information Sheet describing the study, and a consent form. People who responded to this information were spoken to by a member of the research team and asked about ethnicity, age, gender, and long COVID status to confirm eligibility.

The authors purposively sampled — as outlined by Patton^38^ — people from Arab, Black, South Asian, or any minority mixed background. Participants were asked to describe their ethnicity, and the exact phrase provided was used to accompany their quotations. We asked participants not only to advise us on their ethnicity according to the UK Census data list, but we also asked people to self-describe their ethnicity to label the data extracts presented in the Results. They also completed a long-COVID checklist (based on the World Health Organization’s [WHO’s] definition of the condition^39^) to confirm that they experienced COVID-19 symptoms (probable or test confirmed) lasting ≥ 12 weeks, which were not explained by another condition and, generally, affected their everyday functioning. Seeking health care or receiving a diagnosis of long COVID was not necessary for study inclusion.

### Data generation

A topic guide (Box 1) directed one-to-one, semi-structured interviews and was modified iteratively as data were generated and analysed. The topic guide was developed from the existing literature and in discussion with the expert advisory group and patient advisory group. The topic guide was modified iteratively as data collection and analysis progressed. Participants provided informed consent prior to the interview. Interviews were conducted online, using video software (Microsoft Teams^®^), with the camera turned on (at least initially to confirm participant identity)^40^ or via telephone.

**Box 1.** Interview topic guide

**Topics:** Experiences of symptoms Impact of symptoms Use of wider support systems Experience of healthcare services or other supports Support and treatment preferences Facilitators/barriers to accessing support What recovery might look like Experiences of stigma, discrimination, and racism in health care Culture, religion, and spirituality

Interviewers were four females from different backgrounds (two were White British, one was White Polish, and one South Asian) and one male of a White Australian background. Interviewers had varying years of qualitative expertise and were unknown to participants. Interviews were conducted in English between June 2022 and June 2023 (average duration of the interview length [mean duration] 1 hour 36 minutes; duration range: 54 minutes to 4 hours 31 minutes). Interviews were digitally recorded, transcribed, and anonymised. Participants received a debrief form (including details of long-COVID support) to be completed within 1 month and a shopping voucher following the interview.

### Analysis

Anonymised interview transcripts were uploaded into NVivo 14 software for coding. The first author read all transcripts and analysed the data iteratively using inductive thematic analysis^41^ and constant comparison.^42^ Interpretation of the findings were discussed with the study authors, patient and expert advisory groups, as well as during two online public stakeholder workshops that were attended by people living with long COVID, carers, researchers, healthcare professionals, and members from long-COVID charities. Stakeholders were recruited by personal invitation, snowballing, long COVID Support and long COVID SOS groups, and social media. A total of 150 people participated in the two workshops.

All authors were involved in debating and developing multiple iterations of the manuscript until finalised.

## Results

### Participant demographics

In total, 31 participants out of 61 advertisement responders, with a range of socioeconomic demographics and experiences (Table 1), were interviewed. The themes developed included:

- symptoms and help-seeking;
- navigating healthcare access; and
- experiences and perceptions of stigma and discrimination.

**Table 1.** Participant (*n* = 31) characteristics

**Characteristic**	***n* (%)**
**Gender**	
Male	15 (48.4)
Female	16 (51.6)

**Age range**	
20–29 years	9 (29.0)
30–39 years	10 (32.3)
40–49 years	6 (19.4)
50–59 years	4 (12.9)
≥60 years	1 (3.2)
Missing	1 (3.2)

**Ethnic background**	
Arab	3 (9.7)
Black	10 (32.3)
South Asian	10 (32.3)
Mixed heritage	6 (19.4)
Other	2 (6.5)

**Occupational background**	
Student or not employed	7 (22.6)
Healthcare sector	4 (12.9)
Educational/professional sector	10 (32.3)
Transport sector	3 (9.7)
Sales/customer services	4 (12.9)
Skilled trade	2 (6.5)
Missing	1 (3.2)

**Social standing** ^a^	
1–3	3 (9.7)
4–7	20 (64.5)
8–10	3 (9.7)
Did not want to answer	4 (12.9)
Missing	1 (3.2)

**Place of residence**	
Southern England	6 (19.4)
London and Greater London	13 (41.9)
Midlands	4 (12.9)
Northern England	3 (9.7)
East England	1 (3.2)
Scotland	1 (3.2)
Wales	2 (6.5)
Missing	1 (3.2)

**Pre-existing condition**	
Yes	15 (48.4)
No	16 (51.6)

**Year of first COVID-19 infection**	
2020	14 (45.2)
2021	11 (35.5)
2022	5 (16.1)
Unknown	1 (3.2)

a *Subjective social status measured using the MacArthur Scale of Subjective Social Status; a higher score represents higher social standing in a person’s community.*

Illustrative quotations are provided to support the analysis. Data extracts are labelled with gender and ethnicity, along with pseudonyms.

### Symptoms and help-seeking

Acute COVID-19 symptom severity varied. Some participants described mild symptoms that were manageable at home, often requiring adjustment to their daily routines. Other participants described more-severe symptoms that resulted in them seeking urgent medical attention and/or left them feeling scared, helpless, and/or debilitated. Although nearly half of participants reported having a pre-existing condition, most described themselves as being relatively healthy previously:

*‘The first day I had a bit of a fever. Umm and then for a couple of days after that I had some difficulty breathing and I had a lot of joint pain and fatigue … I’m usually quite healthy and I like do exercise and stuff, so that was the first for me, like not being able to breathe properly … so I kind of just had to lie down or just sit down …’*

(Aarya, female, Bangladeshi)

Participants described their slow realisation that they were not recovering after an acute COVID-19 infection. They either seemed to take a long time to recover, or felt better and then experienced relapsing symptoms:

*‘… I just thought like “ohh I need to start going to bed earlier or I must just be tired from work” … I never thought it was like a disease. But then it just started getting worse and worse, and then it started getting to a stage where like I was really noticing it …’*

(Hassan, male, South Asian and White heritage)

They also described ongoing symptoms relating to different parts of their body, with some also describing disruptions to their family lives, daily routines, and/or their employment. Some symptoms were so severe they led to participants being unable to carry out ordinary daily tasks and seeking medical attention:

*‘My body just kind of went “woof” … I couldn’t stay awake for a whole day … I would get vertigo … the most basic things just became impossible … I didn’t have the energy to … prepare a meal … brush my teeth or stand up in the shower … just all sorts of really basic bits of self-care or, like, home kind of management would just not happen … I stayed off work … for just over a year … I started to get just like mental fatigue as well … trying to concentrate at the computer … would just make me feel nauseous for the rest of the day and really tired …’*

(Abi, female, Black Asian)

Sometimes, a lack of awareness of long COVID as a condition, or that support might be available for their symptoms, was related to delayed help-seeking:

*‘People aren’t aware that it’s* [long COVID] *really a thing, or that what they’re experiencing might be linked to it … I would have asked for help if I knew there was treatment available … if there was something that I knew existed …’*

(Aarya, female, Bangladeshi)

Other participants described delaying help-seeking, because of cultural barriers, such as believing they need to look after others before themselves. Some made comparisons with others who were worse off and, as such, felt they were not a candidate for care,^43^ which delayed them seeking help:

*‘… I’ve always found it hard to make my needs known … and I think that’s a cultural upbringing thing … looking after other people first …’*

(Naomi, female, Asian)

*‘… I just live with it, there’s no support for it. I just live with it, and I feel it’s not as bad as other people … my symptoms are mild comparing with others because it is long-term …’*

(Jamila, female, Arabic)

### Navigating healthcare access

When seeking health care for symptoms, participants commonly came up against complex and inflexible primary healthcare systems, making it difficult to access a GP appointment:

*‘It’s been quite negative because every time we try to call the GP, or like we tried to call 111, it was like, it was basically just really, really hard to reach someone … and once you get through, they’d be like “call this person or try this other service” so we’re just kind of going in a roundabout …’*

(Aarya, female, Bangladeshi)

They also described how their symptoms — such as, breathlessness, memory, or concentration difficulties — hindered access and made it difficult to speak to a doctor and communicate symptoms:

*‘It was really hard to even have a phone call with that doctor because I kind of have to … write your thoughts down beforehand, my mum suggested that.* [She said:] *“Write it all down so you don’t forget.”’*

(Naomi, female, Asian)

Many participants described how the barriers associated with navigating primary healthcare systems (that is, getting an appointment and difficulties communicating their symptoms or concerns) meant they needed the help of wider systems of support, such as family members:

*‘If not for some assistance that my wife’s brother could actually* [provide]*, I don’t think I would have been admitted* [to hospital]*, because they were inferring that we could just go back home … The help that I got from a GP was through my wife’s brother … he called the GP in for me … so he was actually just concerned.’*

(Peter, male, Black British)

GPs were recognised by participants as gatekeepers to wider support for long COVID. However, notably, participants spoke about the need to become ‘your own doctor’. They described how they felt they had become experts in their condition from their own research and experience of navigating ongoing or further support; this, typically, involved participants lobbying for investigations, medication, or referrals to specialist services:

*‘I’m having to chase my own results and sort of be hypervigilant … I’m constantly having to test my results … speak to my GP to see if they’ve got it* [results]*, to see if they want to do something about it …’*

(Meera, female, British Indian)

A few participants saw their GP as excellent and described positive interactions. They described receiving recognition and being believed, which were crucial to their good mental health, and feelings of trust and safety:

*‘My GP is amazing … I’ve seen two separate GPs in the past couple of years … I feel so, so lucky to have this amazing surgery … most doctors I’ve met have not taken that approach, but my GP absolutely does. So, he really, really listens … I totally trust him …’*

(Adunola, female, Black African)

However, a more-common experience described by participants was health care not meeting their needs. Many participants did not feel they had support from GPs (or other primary care services); some put this down to GPs having limited awareness and knowledge of long COVID, as well as limited time or resources, and/or available clinical treatments or investigations:

*‘I’d say the NHS, the GPs, specialists, and A&E* [accident and emergency] *have been, I don’t know, if they are well enough informed or can’t keep up with the research or* [are] *restricted by what they can do. Apart from ruling out any other problems, I have received pretty much zero support from them. All the advice has been terrible …’*

(Antonio, male, Latino)

Most participants said GPs seemed to have little empathy for their suffering and seemed to have little interest in engaging with long COVID:

*‘My GP never really thought that she needed to do anything really. Basically … she just said, “oh well, you’ll get better” …’*

(Hassan, male, South Asian and White heritage)

*‘It’s almost as if they would rather not know because they don’t know how to fix* [long COVID]*.’*

(Meera, female, British Indian)

Referrals to onward care were perceived as difficult to obtain by those who attempted to access this support. Barriers to accessing secondary care (including long-COVID specialist clinics) included a lack of awareness from the participants about available services, as well as primary care providers not being aware of the referral processes involved:

*‘I’ve been passed from pillar to post, chasing referrals with the hospitals because either my GP has done it on the wrong form, or they’ve done it incorrectly.’*

(Samira, female, British Indian)

*‘I didn’t know there is a special clinic that deal with after COVID* [long COVID]*, the problem, no- one mentioned anything.’*

(Jamila, female, Arabic)

Participants generally needed to be persistent in requesting referrals to onward care:

*‘I reported it to my GP … but I had to really push before my GP referred me to the long- COVID clinic.’*

(Deepti, female, British Indian)

Many times, participants reflected on their disappointment when they accessed long-COVID clinics. Care did not seem to match participants’ symptoms and, as such, support was perceived as unhelpful:

*‘… a cardiologist give me a medical advice to drink fruit juice and try not to think about being ill … I had a doctor tell me that I have to stop resting and actually push through exercise to feel better … I’ve been to a long-COVID clinic, which was basically one doctor — he’s a pulmonologist — and he said because I can breathe properly, he can’t really help me with anything else …’*

(Antonio, male, Latino)

Some participants were able to access appropriate support in terms of being believed and/or referred for more in-depth investigations:

*‘*[The doctor said] *“I believe everything you’re saying and we’re finding long haulers have this post-inflammatory issue going on and all these other issues that tests are coming back normal, but there’s something very wrong going on here” … so, he sent me for another MRI* [magnetic resonance imaging] *scan, but with a more specialised perfusion … that came back showing I had myocarditis, heart inflammation …’*

(Ahmed, male, Chile, White heritage)

One participant described this was only after doing intensive research and lobbying for the referral, while another described joined-up care:

*‘The person that got in touch with me was, for the* [long] *COVID clinic, an occupational therapist. She has been an absolute godsend … I’ve described her as the linchpin in all this because she’s collating everything, she’s going back to the multidisciplinary team and she’s just helping me emotionally as well … fantastic.’*

(Samira, female, British Indian)

### Experiences and perceptions of stigma and discrimination

Participant experiences of health-related stigma^29^ were evident in narratives of interactions with others. Participants reflected on a lack of awareness of long COVID being linked to the stigma around the condition:

*‘They don’t know what long COVID is … if they knew about it, they … will be less quick to judge … it’s not really … talked about … when you treat a particular illness or sickness, it’s expected that you feel better completely and no, like, lingering symptoms … something like long COVID is really difficult for people to understand …’*

(Winston, male, Black/Caribbean)

Participants described how others treated them differently because of their long-COVID symptoms:

*‘So many people are still not aware of the post* [long] *COVID. They’re still thinking it’s COVID- 19 as it were, and many people are still scared of contracting the COVID, so you need to, like, explain to them … look, this is not COVID, this is just … not something to be scared of.*’

(Jevaun, male, African Caribbean)

One participant described how they did not want to connect their ongoing symptoms with COVID-19:

*‘I think I’ve left COVID- 19 behind … I don’t want to link it* [symptoms] *to COVID- 19, but I don’t want to believe it* [is] *still because of COVID- 19 that I’m still having these symptoms …’*

(Char, female, Black African)

Consequently, participants needed encouragement from personal networks to believe that their symptoms might be linked to COVID-19, were real, or were worthy of accessing health care:

*‘I need to be sure … I think I’m okay talking to someone who is close to me, will actually give me a talk to guide me, and then, I would be able to access the doctor, and then, know what the issue actually is, so I can be free.’*

(Char, female, Black African)

However, seeking support from support systems, such as family members, was not always helpful. Some participants anticipated stigma; they described the need to hide their symptoms, as they expected prejudice, and this was especially reported by females:

*‘In our culture and community, people don’t understand. You always have to pretend* [not to have symptoms] *… they would make nasty remarks about me …’*

(Manya, female, South Asian)

Some participants described how they hid psychological symptoms or resisted mental health labels (for example, anxiety), which meant they could not talk to others or discuss the full range of symptoms they were experiencing, as they worried how they would be perceived by others, including healthcare professionals. They anticipated being discredited, dismissed, or not receiving adequate health care:

*‘It’s also just hard to talk about because, yes, you do think “gosh, am I losing my mind?”, and you wonder how people will perceive you because of that.’*

(Farah, female, Middle Eastern heritage)

*‘I mean, because of my experiences, I started denying anxiety. I don’t have anxiety because I realised, once anxiety comes into the mix, you get dismissed. So, that was another thing. I just started saying “no, I’m not anxious about anything at all, nothing”… ’*

(Paulette, female, British Black Caribbean)

Many participants anticipated experiencing negative healthcare encounters because of previous experiences in health care. For instance, a common experience described by participants was having their accounts discredited or dismissed by healthcare professionals:

*‘So, I didn’t* [see healthcare professionals for long-COVID symptoms] *that’s probably because I just assumed they would just say, like, “just rest” or whatever. Yes, so I would say that my past experience with the dismissiveness, so there’s no point trying to address this.*’

(Anjali, female, British Indian)

Participants attributed being treated differently to certain characteristics about themselves, such as ethnicity, gender, mental health, and body type (intersectional stigma).^28^^,^^30^ They considered the treatment they received (for example, gaslighting) to be linked to their ethnicity and racism in particular:

*‘I mean, you’re going for help, so people ignoring you and you feel like you’re not feeling pain, possibly because you’re a Black person, you’re lying …’*

(Paulette, female, British Black Caribbean)

*‘… I don’t like being in the hospital, especially White hospital … there is a way you are being treated … maybe because you have been seen as, probably, an ethnic minority …’*

(Peter, male, Black British)

Other participants believed that healthcare professionals saw other markers, such as mental illness or their weight, as the cause of their symptoms and that this could get in the way of receiving treatment:

*‘They always pull it back to your weight, because it seems to be the easiest thing and the most obvious thing for them to comment on … the discriminatory act comes into it because it’s almost like saying “well, you are responsible for your weight, therefore it is on you that this has happened”.’*

(Samira, female, British Indian)

Several female participants experienced intersectional stigma in particular; they described the difficulties of having their symptoms taken seriously as healthcare professionals might think they were overreacting because they were female, or that long COVID itself might be discredited as it is more common in women:^2^

*‘I don’t have any evidence of it, but I sometimes feel like if I was a man I would be taken more seriously. I’m not sure why but I’ve had healthcare professionals … questioning my symptoms and they’re trying to convince me or suggest that my symptoms are actually things like anxiety or, you know, being overly worried about COVID or that I’m exaggerating my symptoms.’*

(Farah, female, Middle Eastern heritage)

Notably, when participants felt discriminated against because of ethnicity, body type, or gender, this reduced their feelings of safety and trust in their health care. This medical mistrust^44^ hindered help-seeking and the quality of care received; some participants only sought health care as a last resort, and some felt anxious or stressed about seeking further healthcare support:

*‘I’ve experienced racism and sexism in my health care … I’m really nervous about going to the doctors now; I will not go unless I am desperate now basically … with my long COVID, I didn’t actually seek help until it got to eight months … and then I am still dismissed …’*

(Deepti, female, British Indian)

*‘I got distrust of medical staff now, especially now, especially as a person of colour. I don’t trust anything they say or do. It* [is] *just experiences after experiences after experiences, time after time.’*

(Paulette, female, British Black Caribbean)

Participants wanted fairer treatment from healthcare professionals; as an example, one reflected on how they wanted to be treated the same as people from a White background:

*‘… that treatment would just be fair … just like everybody else is treated … but I feel my people should also be, my people should also be treated in that way.’*

(Peter, male, Black British)

Participants reported that, in order for things to improve for people ‘like them’, their suffering needed to be taken at face value by healthcare professionals, which included recognition that, regardless of how they presented their symptoms, it was equal to that of White patients:

*‘Not be gaslighted, not be fobbed off, not be pushed from pillar to post. Just to be treated. If I come in with symptoms, treat me the same you would as if it was a Caucasian person.’*

(Anthony, male, Black American)

## Discussion

### Summary

This study explored the lived experiences of long COVID in people from ethnic minority groups, substantially adding to the literature on long-COVID experiences among these populations.^11^ Participants described a range of reasons why they delayed help-seeking, such as being unaware of long COVID as a condition or the available support, or not feeling as though they were a candidate for care. Accessing primary care involved barriers, such as inflexible or complex systems, symptoms hindering communication with doctors, and a lack of interest in long COVID in primary care. Secondary care or specialist long-COVID clinics could require persistence in asking for a referral and/or chasing one. Long delays in accessing specialist long-COVID services were common. Many participants expressed disappointment with the care they ultimately received in both primary and secondary health care.

Participants reported experiencing additional burdens when accessing and receiving health care for long COVID. As an example, they described experiencing health-related stigma that intersected with ethnicity, gender, body type, and mental health status. Participants who wanted their suffering to be recognised also wanted fairer treatment. Experiences of stigma and discrimination reduced participants’ trust in health care and were seen as clear barriers to accessing good care.

### Strengths and limitations

A key strength of the study is participant diversity in terms of ethnic minority background, equal representation of males and females, socioeconomic status, and residence across the UK. However, there were few representations from older age groups (≥ 60 years). Experiences were from a range of ethnic minority groups and, as such, may not be representative of a single ethnic minority.

Another key strength of the study was involvement from a diverse patient advisory group in terms of ethnicity and residence across the UK in all stages of the research, as well as involvement from an expert advisory group at key stages of the research. This resulted in research that contributed to a wider understanding of long COVID in diverse groups. Criteria for long COVID was participant self-disclosed, based on the WHO’s clinical case definition of post-COVID-19 condition, which meant that people who had not accessed health care for their symptoms could still be involved.^45^ Involvement and engagement with the advisory groups and stakeholder workshops guided the interpretation of findings, providing support to the analysis. Experiences were not directly compared with those of people from White backgrounds; to better understand the specific challenges faced by people from ethnic minorities (beyond their own perceptions of the issues), a comparative analysis would be needed.

### Comparison with existing literature

The current findings echo research showing ethnic inequalities in accessing health care.^46^^,^^47^ However, they add considerable nuance to the understanding of why people from ethnic minority backgrounds are less likely to access primary care for long COVID (as identified by Magadi *et al*)^20^ and why, when primary care services are accessed by these groups, they report less satisfaction with care received;^24^ this complements what has been reported about experiences from people from White backgrounds.^8^^–^10^,^^13^

Despite specialist long-COVID services having been established across England,^48^^,^^49^ some study participants were unaware of services or experienced difficulties accessing them; this finding is consistent with an inadequate representation of patients from ethnic minorities in long-COVID clinics.^21^^,^^22^ Moreover, some participants expressed dissatisfaction with care received from long-COVID clinics, which is consistent with the experiences of disadvantaged groups reported by Baz *et al*.^11^

Typical experiences for people living with long COVID, regardless of ethnic background, include difficulties accessing health care, not being believed by their doctor, lack of understanding or knowledge of long COVID from their doctor, overstretched and complex health systems, and needing to advocate to get care.^8^^–^10^,^^12^^,^^13^ People from ethnic minority backgrounds experienced additional burdens associated with long COVID, particularly linked to fearing prejudice from others (within the community and health care); this often resulted in delayed help-seeking and them denying aspects of their condition, such as psychological symptoms, which is consistent with previous research indicating that stigma can be a particular barrier to disclosing mental health problems for people from ethnic minorities.^28^^,^^50^

Participants also experienced anticipated stigma when care expectations were based on previous negative healthcare encounters, intersecting with racism, body type, or gender, which also delayed help-seeking, and they believed they received less-favourable treatment compared with people from White backgrounds, and connected this to their ethnicity (intersectional stigma). These findings are consistent with racism contributing to ethnic inequalities in healthcare access, as well as dissatisfaction with care received.^51^^,^^52^ In all, the various kinds of discrimination experienced by participants resulted in reduced trust in health care; such ‘medical mistrust’ is often seen in ethnic minority groups accessing care for long COVID^11^ and other conditions, including mental health and maternity care.^53^^,^^54^ Consequently, people from ethnic minority backgrounds, in general, may feel less deserving of receiving care than other groups.^43^ When care was sought, many participants were dismissed, which confirmed their expectations — and, for some, intensified the emotional burden — of living with long COVID.

### Implications for research and practice

It is unknown whether participants migrated to the UK or English was their first language, which may have affected their accessing health care; this could be explored in future research. Primary care is the first point of contact for accessing support for long COVID;^55^ it has a crucial role in reducing health inequalities for underserved groups,^56^ including people from ethnic minority groups, who are less likely to access these services.^20^ The findings presented here suggest that this may be due to health-related and intersectional stigma.^28^^–^30 People fear poor care or discrimination, which reduces trust in health care. Dismissing or discrediting people’s suffering can result in them feeling discriminated against because of their ethnicity, body type, or gender, thereby contributing to their not receiving adequate care. There is a need for people to be believed, and have symptoms recognised, by clinicians. Moreover, there was a lack of awareness of long-COVID symptoms or available support services, as well as participants not feeling worthy of receiving care.^44^ However, support from people’s social networks may facilitate help-seeking behaviours.

Findings from this study have informed development of a long-COVID support tool available at https://long-covid-care.org.uk/ (led by STIMULATE-ICP)^57^ to help raise awareness of long-Covid symptoms and available support for people who may have long-COVID symptoms and healthcare professionals. NICE’s guidelines on managing long COVID^4^ were updated in 2024, but lived experiences from diverse groups are still not represented; the current findings should be considered when managing people with symptoms (be they probable or confirmed) of long COVID.

Participants’ accounts of being dismissed, disbelieved, or discredited speaks of epistemic injustice (that is, undermining or not acknowledging people’s knowledge or experiences)^58^ when seeking health care for long COVID,^59^ which is especially related to racism and sexism.^60^ Key recommendations from NICE’s long-COVID guidelines are to believe, listen to, and legitimise people presenting with symptoms indicating long COVID.^4^ The findings presented here further highlight the importance of these recommendations for people from ethnic minority backgrounds, who face additional challenges when seeking care and support for long COVID. Awareness-raising strategies to reduce stigmatising beliefs or behaviours of healthcare professionals are needed to deliver care that is non-discriminatory. Empowering GPs to be welcoming and open^61^^,^^62^ when presented with limited treatment options and limited understanding of a condition, such as long COVID, is needed. In the case of long COVID, a focus on listening, believing, and empathising^9^ will help healthcare professionals connect with patients, thereby improving patient experiences of warmth in healthcare encounters — something for which people from ethnic minority^63^ backgrounds are calling for in health care. This is a crucial step for patients to gain trust in healthcare professionals and services, and may go some way to restoring epistemic justice in health care.^60^

Further analyses related to the wider data from this study will be published elsewhere, led by the second author; this further analysis focuses on the need for recognition and legitimacy for long COVID, how medical ambivalence manifests for patients, and the important role of wider sources of support.
